# Current challenges in the clinical management of sexually transmitted infections

**DOI:** 10.1002/jia2.25347

**Published:** 2019-08-30

**Authors:** Henry JC de Vries

**Affiliations:** ^1^ STI Outpatient Clinic Infectious Diseases Department Public Health Service (GGD) Amsterdam Amsterdam The Netherlands; ^2^ Department of Dermatology Amsterdam UMC Amsterdam Institute for Infection and Immunity (AI&II) University of Amsterdam Amsterdam The Netherlands

**Keywords:** sexually transmitted infections, syphilis, gonorrhoea, antimicrobial resistance, lymphogranuloma venereum, *Mycoplasma genitalium*

With the emergence of HIV in the 1980s, the first people diagnosed with AIDS were treated by a variety of medical specialists. For some, like internal medicine specialists, dealing with a sexually transmitted infection (STI) was a new aspect in their patient contact. For others, like genitourinary medicine specialists and dermatologists, the many HIV‐related internal medicine‐related morbidities posed a challenge. Frequent interdisciplinary consultation made clinical care for people living with HIV/AIDS a multidisciplinary endeavour right from the start.

AIDS was first diagnosed in men who have sex with men (MSM) who had had multiple previous STIs 1. MSM were considered a key population both for HIV and STIs early on. Yet the HIV epidemic had a dramatic effect on adherence to safe sex measures and, as a result, the incidence of bacterial STIs declined rapidly.

With the availability of effective antiretroviral therapy (ART) since 1996, HIV was no longer a deadly infection for those with access to medication. People with HIV on ART could live healthy lives, including having sex. This phenomenon, coined “treatment optimism,” resulted in a rise in bacterial STIs, especially among HIV‐positive MSM 2.

The intertwining of the HIV and bacterial STI epidemics highlights that, to be truly effective, the response to HIV and other STIs should not be echeloned. People living with HIV (PLHIV) are affected disproportionally by STIs, and individuals with STIs are more susceptible to HIV acquisition. This applies especially to MSM, transgender persons and (female) sex workers. In this viewpoint, five current issues of concern in the clinical management and prevention of STI and HIV are discussed. Although the main focus here is on MSM, this does not imply that the STI burden in heterosexual men and women is not substantial.

## Biomedical interventions for HIV and risk compensation

Concerns have been raised that the treatment as prevention (TasP) paradigm for HIV‐positive people and pre‐exposure prophylaxis (PrEP) for HIV‐negative people will induce more risky sexual behaviour, thus increasing the incidence of other STIs 3. This phenomenon is sometimes called risk compensation, where one perceives that antiretrovirals are protective against HIV transmission. However, increases in sexual risk have antedated the implementation of TasP and availability of PrEP 3. The increasing practice of condomless sex and the transmission of STIs and HIV among MSM began after effective antiretrovirals became widely available in 1996, when HIV was no longer considered a deadly disease 4.

Preliminary indications of risk compensation in PrEP demonstration projects and observational studies are conflicting 5, 6, 7, 8, 9. This discrepancy might arise from decreased onward transmission of STIs due to more frequent STI screening, whereas ascertainment bias may increase STI detection. Since the maximum follow‐up time in the published studies was less than two years, it might be too short to observe risk compensation at this time. All in all, HIV clinicians should be prepared for increasing numbers of patients with STI co‐infections. In some regions (such as continental Europe), STI and HIV care are fragmented and offered at different sites; managing co‐infections in PLHIV can be especially challenging in these regions, and integrating care should be considered.

## Emerging STIs

Around the turn of the century, unusual outbreaks of STIs were encountered in PLHIV. Lymphogranuloma venereum (LGV) 10 and hepatitis C (HCV) 11 were emerging STIs that were, by far, mostly diagnosed in HIV‐positive MSM living in metropolitan areas, and engaging in risky behaviour such as fisting.

LGV is caused by an invasive variant of *C. trachomatis* and causes a severe and destructive infection in the anogenital region. The true magnitude of the LGV epidemic is underestimated because of a scarcity of routine screening and surveillance efforts, as well as the considerable proportion of presentations that are asymptomatic 10. Moreover, preventive measures to reduce transmission are hindered, and they will be, as long as the mode of transmission is not fully understood. The overrepresentation of anorectal versus genital LGV infections (15:1) suggests that other modes of transmission occur apart from anal sex.

Among the first people diagnosed with LGV, alarming numbers of HCV co‐infections were also diagnosed 12. Sexual transmission of HCV was subsequently identified among HIV‐positive MSM. Until then, it had been considered to be a blood‐borne disease 11. Most recently, transmission of HCV from HIV‐positive MSM to HIV‐negative MSM who use and intend to use PrEP has been observed 13. Suggested causes are “sero‐mixing” (sex between serodiscordant partners) and risk compensation. As with LGV, the sexual transmission of HCV is not fully elucidated, which hinders preventive measures.

Many HIV‐positive MSM form core STI transmitters and often take a central position in sexual networks. These networks expand globally, as demonstrated for HCV 14 and LGV 15. For HIV care specialists, this stresses the importance of close ties with public health institutions, continued global surveillance and early warning measures.

## Multiplex diagnostic nucleic acid amplification tests

Nucleic acid amplification tests (NAATs) have revolutionized the diagnostic process for STIs. Traditionally, STI screening relied on direct light microscopic visualization, cultivation of pathogens and serology. Although these tests modalities are characterized by high specificity, sensitivity was often low. The amplification of pathogenic DNA or RNA proved extremely useful for the development of highly sensitive and specific tests 16. Although still way too expensive for most low‐ and middle‐income countries, the ease of use in sample collection for NAATs has led to wide implementation in high‐income countries.

NAATs allow the integration of STI screening outside the traditional STI outpatient clinic setting, for example, in the context of routine HIV care. Moreover, NAATs offer options of (patient) self and home collection, thus substantially simplifying STI screening. Commercial parties increasingly launch NAATs that can diagnose multiple pathogens in a single specimen. This can have cost benefits in the elucidation of the causative organism of an STI‐related syndrome, such as urethritis, vaginal discharge or genito‐ulcerative disease.

Yet there is a downside that can induce over‐treatment of organisms considered to be non‐harmful or clinically irrelevant. *Mycoplasma genitalium* is one such organism, whose clinical relevance, especially in asymptomatic people, is debated 17, 18, 19. *M. genitalium* has been associated with urethritis in men, and most guidelines recommend testing only in symptomatic people. Moreover, the treatment of *M genitalium* is increasingly complicated by antimicrobial resistance. The advent of commercial multiplex NAATs containing *M. genitalium* as the target puts clinicians and microbiological laboratories in a treatment dilemma. When positive results are found in asymptomatic individuals, over‐consumption of antibiotics will only increase antimicrobial resistance, which is another emerging threat in the management of STIs.

## Antimicrobial‐resistant gonorrhoea

With 78 million new cases of gonorrhoea globally, gonorrhoea is the second most prevalent bacterial sexually transmitted infection worldwide 20. Persistent infections may cause severe genital and reproductive tract inflammation and damage, like pelvic inflammatory disease, ectopic pregnancy, epididymitis and infertility; gonorrhoea also increases the transmission of HIV 21. The World Health Organization's (WHO's) first general global report on antimicrobial resistance, published in 2014, revealed that antibiotic resistance is no longer a prediction for the future; it is happening right now, across the world. This is even more worrisome since no major new types of antibiotics have been developed over the past 30 years 22. Moreover, this report specifically mentions treatment failures due to resistance to extended spectrum cephalosporins (the last‐resort treatments for gonorrhoea) in 10 countries, and decreased susceptibility in 36 countries. Thus, gonorrhoea may soon become untreatable. There are some promising antibiotics in the pipeline, such as zoliflodacin 23 and gemifloxacin, which have not reached market yet 24.

## Shortages of out‐of‐patent antibiotics

In 2017, a global shortage of benzathine penicillin G (BPG), the first‐line treatment option for syphilis, was reported. The largest indication for BPG is rheumatic heart disease; syphilis accounts for only 1% of BPG prescriptions. BPG is the only option considered safe for pregnant women in the prevention of congenital syphilis 25. Since the profit margins of BPG are small and the production costs are high, the active pharmaceutical ingredient was produced in only three factories, all based in China. This has dramatically increased the stock‐out risk. Recently, two of the manufacturers terminated their production due to governmental regulatory and environmental issues.

WHO has recognized BPG as an essential medicine at high risk for stock‐out 26. It has invited manufacturers to apply for WHO pre‐qualification to ensure acceptable quality, safety and efficacy standards of BPG supplied by international agencies (for example, the Global Fund to Fight AIDS, Tuberculosis and Malaria).

From a demand perspective, national‐level BPG forecasting and procurement systems should be strengthened and appropriate treatment of syphilis should be prioritized. Since the first‐line treatment options for chlamydia, gonorrhoea and trichomoniasis are also off‐patent antibiotics, future shortages can be envisioned here as well.

## Concluding remarks

Since key populations often overlap each other, it is necessary to de‐silo STI and HIV care. In the UK and most former Commonwealth countries, HIV and STI care are fully integrated in sexual health clinics. From a quality of care perspective, this seems to be most ideal: a “one‐stop shop” setting where patients are holistically managed. From a public health perspective, integrated care offers the opportunity to address contact tracing and preventive interventions for both HIV and STI key populations.

Yet in many regions, STI and HIV care are still offered by separate medical specialties in separate settings. As a result, at the least, resources are wasted. More often though, fragmentation of care leads to delays, non‐adherence, loss to follow up and, onward propagation of infections. It is important that these settings work towards desegregation of care and adopt the format of integrated sexual health clinics where screening, treatment, follow up and preventive interventions are offered to patients and to key populations.

PrEP has proved to be a highly effective tool against ongoing transmission of HIV. Yet, PrEP also offers opportunities to assess new STI prevention strategies. The currently developed NAATs promise faster availability of results and will become true point‐of‐care tests that can be integrated into routine HIV care. This will enable infection management (including counselling, treatment and contact tracing) while the person waits during a single consultation, further limiting ongoing transmission. Treatment of STIs will remain a point of concern in the coming years, either due to emerging antimicrobial resistance or drug shortages.

## Competing interests

The employers of the author have received money or goods from: Medigene, Gilead, MSD, Abbott, Janssen and Novartis for his research, presentations and advisory activities.

## Authors’ contributions

HdV wrote this manuscript.
